# Hyperspectral image spectral-spatial classification via weighted Laplacian smoothing constraint-based sparse representation

**DOI:** 10.1371/journal.pone.0254362

**Published:** 2021-07-13

**Authors:** Eryang Chen, Ruichun Chang, Ke Guo, Fang Miao, Kaibo Shi, Ansheng Ye, Jianghong Yuan

**Affiliations:** 1 College of Geophysics, Chengdu University of Technology, Chengdu, China; 2 School of Electronic Information and Electrical Engineering, Chengdu University, Chengdu, China; 3 Geomathematics Key Laboratory of Sichuan Province, Chengdu University of Technology, Chengdu, China; 4 Key Laboratory of Pattern Recognition and Intelligent Information Processing of Sichuan, Chengdu University, Chengdu, China; 5 Digital Hu Line Research Institute, Chengdu University of Technology, Chengdu, China; 6 School of Intelligent Engineering, Sichuan Changjiang Vocational College, Chengdu, China; National University of Sciences and Technology (NUST), PAKISTAN

## Abstract

As a powerful tool in hyperspectral image (HSI) classification, sparse representation has gained much attention in recent years owing to its detailed representation of features. In particular, the results of the joint use of spatial and spectral information has been widely applied to HSI classification. However, dealing with the spatial relationship between pixels is a nontrivial task. This paper proposes a new spatial-spectral combined classification method that considers the boundaries of adjacent features in the HSI. Based on the proposed method, a smoothing-constraint Laplacian vector is constructed, which consists of the interest pixel and its four nearest neighbors through their weighting factor. Then, a novel large-block sparse dictionary is developed for simultaneous orthogonal matching pursuit. Our proposed method can obtain a better accuracy of HSI classification on three real HSI datasets than the existing spectral-spatial HSI classifiers. Finally, the experimental results are presented to verify the effectiveness and superiority of the proposed method.

## Introduction

Remote sensing is of paramount importance for several application fields, including environmental monitoring, urban planning, ecosystem-oriented natural resource management, urban change detection, and agricultural region monitoring [1, 2]. Hyperspectral images (HSIs), whose structure consists of two spatial dimensions and one spectral dimension [3, 4], are generally characterized by hundreds or thousands of continuous observation bands throughout the electromagnetic spectrum with high spectral resolution in the field of remote sensing. The abundance of spectral information in HSI provides an opportunity for the precise classification of ground objects [5, 6]. HSI classification, as one of the main challenges in remote sensing technology, has opened new avenues in remote sensing [7–10]. As a powerful image-processing tool, the support vector machine (SVM) [11–14] and sparse representation (SR) model [15, 16] and its derivative model have attracted much attention for HSI classification [17–20]. However, the noise and mixed spectral information in HSI cause several theoretical and practical challenges for pixel-wise classification [21–23].

A large number of spatial-spectral combined HSI classifiers have been developed in recent decades to incorporate spatial information in the classification. Reference [24] proposed an image patch distance (IPD) that uses the observed pixels and spatial neighbors to measure the pixel patch-wise similarity. Reference [17] presented a joint sparse representation (JSR) model, which first defined a local region of fixed size for each test pixel. Reference [25] reported that a multiscale adaptive sparse representation (MASR) model, which considers the regions of different scales for classification, can further improve classification performance. Reference [26] showed a class-dependent SR classifier for HSI classification, which can effectively combine the SR and k-NN classifiers models in a class-wise manner to exploit both the correlation and Euclidean distance between training and test data. As the traditional joint k-NN algorithm holds, the weight of each test sample in a local region is identical, which is not reasonable because each test sample may have different importance and distribution. To solve this problem, Reference [27] recommended a weighted joint nearest neighbor and sparse representation method, named WJNN-JSR, and can achieve better performance than several traditional joint k-NN methods. More recent HSI classification techniques can be found in references [28–35]. With respect to the above descriptions, it can be concluded that all these methods ignore the boundary information of different features in the HSI. The common shortcomings hindered the achievement of more satisfactory classification accuracy.

Motivated by the above-mentioned discussions, by combining the spectral and spatial information, we propose a new classification algorithm for HIS, which is termed the weighted Laplacian smoothing constraint-based sparse representation (WLSC-SR) classifier. The primary contributions of this study are as follows.

1. Inspired by the existing ordinary vector Laplacian, a smoothing-constraint weighted Laplacian vector is constructed, which consists of the interest pixel and its four nearest neighbors through their weighting factors [36–38].
2. By forcing the weighted Laplacian vector of the pixel of interest to be zero, a new large block sparse dictionary for sparse representation is developed.
3. In contrast to several earlier studies, the boundary characteristics of the HIS were fully used.

Experiments on three real HSI datasets were conducted and compared with several state-of-the-art spectral-spatial HSI classification classifiers to evaluate the performance of the proposed WLSC-SR method. The results show that WLSC-SR can substantially improve the accuracy of the HSI classification.

### Related works

In this section, we outline the basic theory for WLSC-SR.

### Sparse representation

The mathematical essence of sparse representation (SR) is signal decomposition under sparse normalization constraints [39–41]. A few atoms with the best linear combination are found in the dictionary to represent a signal using the super-complete dictionary of redundant functions as the basis function. It is demonstrated that the HSI pixel can be represented as a linear combination of training pixels from all classes by an unknown test.

Let *y* ∈ *R*^*h*×*l*^ be a test sample in HSI, the label set of the whole training set be *T* = {1,2,3, ⋯, *s*}, where *h* denotes the spectral dimension of the HSI, and *s* is the number of training samples. Therefore, a dictionary *D* ∈ *R*^*h*×*s*^ can be constructed using the spectrum sets of *y* and *T*. Each base of the redundant dictionary *D* is called an atom. Therefore, *y* can be represented by a linear combination of atoms of *D*. However, a linear combination is unlikely to be unique. The sparsest coefficient can help us find a better linear combination. Assuming that there is no noise in the HSI, then the SR model of the clean sample *y* is defined as:

$$\begin{array}{l} min\;{\left\|\alpha \right\|}_{0}\\ s.t.\;y=D\alpha \end{array}$$ (1)

However, there must be noise in the HSI, and the SR model for noisy data can be defined as:

$$\begin{array}{l} min\;{\left\|\alpha \right\|}_{0}\\ s.t.\;{\left\|y-D\alpha \right\|}_{2}\leq \sigma \end{array}$$ (2)

Using the Lagrangian multiplier method, the SR model can be regularized as:

$$min\;{\left\|\alpha \right\|}_{0}+\frac{\gamma }{2}{\left\|y-D\alpha \right\|}_{2}^{2}$$ (3)

where *σ* is the error tolerance and *γ* denotes the regularization parameter.

Generally, the orthogonal matching pursuit (OMP) or simultaneous orthogonal mutation pursuit (SOMP) algorithm is used to calculate formula (3). When the original signal is atomic, the OMP can be unified as the SOMP. The SOMP is selected according to the characteristics of the WLSC-SR algorithm.

### Image patch distance

In hyperspectral imagery, the pixels within a small neighborhood usually consist of similar materials whose spectral characteristics are highly correlated.Based on this fact, the image patch distance (IPD) exploits the observed pixels and corresponding spatial neighbors to measure the pixel patch-wise similarity.

For the observed pixel *x*_*ij*_, its *ω*^2^ neighbors in the *ω* × *ω* spatial neighborhood can be defined as:

$$\begin{array}{l} \Omega (x_{i,j})=\{x_{st}:s=i-r,i-r+1,\cdots ,i,\cdots ,i+r;\\ \;t=j-r,j-r+1,\cdots ,j,\cdots ,j+r\} \end{array}$$ (4)

in which r = (*ω* – 1)/2.

Let *a*_*l*_ and *b*_*l*_ be the *l*th elements of the pixel sets *Ω*(*x*_*ij*_) and *Ω*(*x*_*pq*_). The distance between *a*_*l*_ and spatial neighborhood *Ω*(*x*_*pq*_) is defined as $d(a_{l},\Omega (x_{pq}))=min_{b\in \Omega (x_{pq})}d(a_{l},b)$, and the undirected distance between two pixels *a*_*l*_ and *b*_*l*_ can be defined as follows:

$$d_{u}(a_{l},b_{l})=max(min_{b\in \Omega (x_{pq})}d(a_{l},b),min_{a\in \Omega (x_{ij})}d(a,b_{l}))$$ (5)

where *d*(⋅) is a spectral similarity function, such as the Minkowski Distance (MD) and spectral cosine distance (SCD).

Then, the similarity between the observed pixels *x*_*ij*_ and *x*_*pq*_ can be defined as follows:

$$\begin{array}{l} d_{IPD}(x_{ij},x_{pq})=\sum_{l=1}^{w^{2}}d_{u}(a_{l},b_{l})\\ \;=\sum_{l=1}^{w^{2}}\{max(min_{b\in \Omega (x_{pq})}d(a_{l},b),\\ \;min_{a\in \Omega (x_{ij})}d(a,b_{l}))\} \end{array}$$ (6)

This spatial-spectral similarity measure combines the spatial and spectral features into distance, which improves the classification accuracy.

### Proposed classifier

The pixels in the HSI dataset are high-dimensional vectors that reflect the spectrums of the ground objects. The spectrum vectors of the same class label are more likely to be similar to those of the different class labels. Based on this assumption, the WLSC-SR method exploits the spatial neighborhood to extract spatial-spectral information.

### Procedure of WLSC-SR

**Fig 1:**
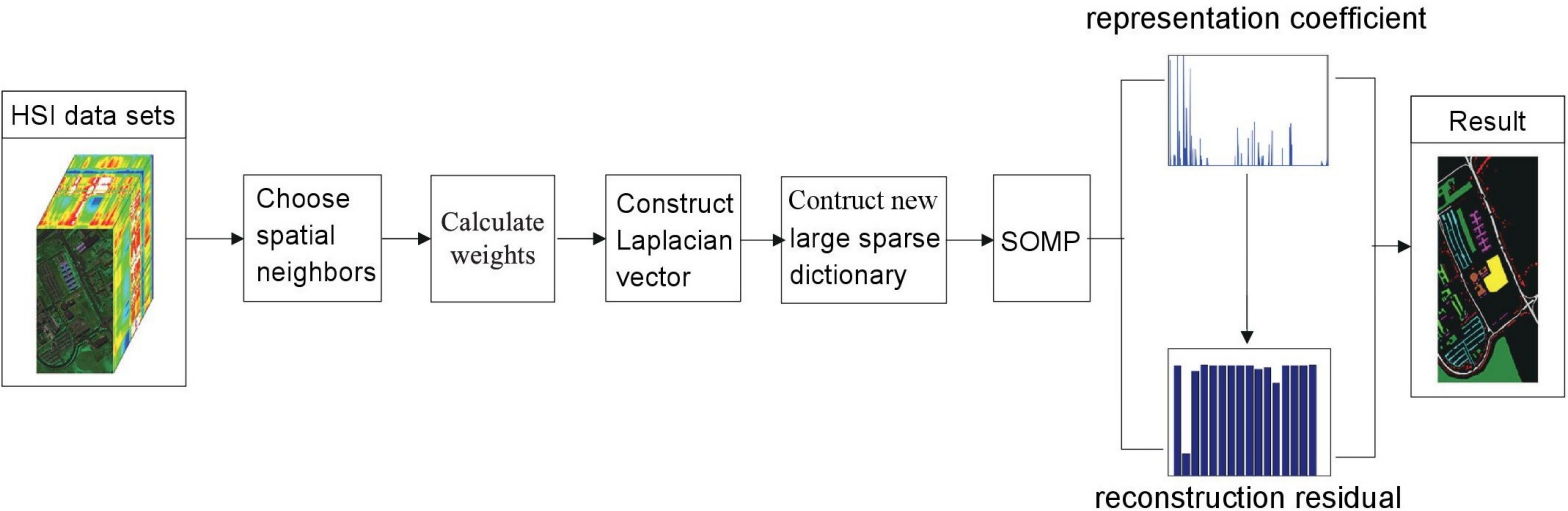
The procedure of WLSC-SR.

The WLSC-SRattempts to construct a smoothing-constraint Laplacian vector, which is forced to be zero. The vector consists of the sparse vector of the pixel of interest and its four nearest neighbors through their weighting factor, by which the aggregation of homogeneous data and the separability of heterogeneous data in HSI can be effectively enhanced. Based on the smoothing-constraint Laplacian vector, a new large block sparse dictionary for SOMP is constructed with six times as many rows and five times as many columns as the original dictionary. Furthermore, the WLSC-SR classifier distinguishes the boundaries of adjacent types of ground objects in HSI, which is beneficial for HSI classification. The procedure for the proposed method is shown in Fig 1.

### Weighted Laplacian smoothing constraint

We assumed that the size of the HSI was *k* × *l* × *h*. The spectrum vector *x*_*ij*_(*h*,*l*) ∈ *R*^*h* × *l*^ represents the pixel in row *i* and column *j* in the HSI. At first, we choose a spatial window parameter *ω*, which is an odd positive integer, and then construct a *ω* × *ω* spatial window with central pixel *x*_*ij*_. At the same time, the HSI boundaries are extended by (*ω* – 1)/2 pixels in a mirror manner, which is convenient for processing pixels at the edge or corner of the image.

We can obtain the IPD between pixel sets *Ω*(*x*_*ij*_) and *Ω*(*x*_*pq*_). However, generally, the IPD method requires a large spatial window to exploit the spatial information in HSI, while the time-consuming steps in the iteration process limit its real applications.

The IPD between pixel sets *Ω*(*x*_*ij*_) and *Ω*(*x*_*pq*_) is replaced by the distance between the central pixels in the pixel sets to simplify the IPD calculation method. Thus, the image patch distance-based center (IPDC) calculation method is defined as follows:

$$d_{IPDC}(\Omega (x_{ij}),\Omega (x_{pq}))=d_{IPD}(x_{ij},x_{pq})$$ (7)

Thus,the weighting factor can be defined as:

$$w_{\left(\Omega (x_{ij}),\Omega (x_{pq})\right)}=exp(-\frac{d(x_{ij},x_{pq})}{t})=exp(-\frac{{\left\|{\tilde{x}}_{ij}-{\tilde{x}}_{pq}\right\|}_{2}^{2}}{t})$$ (8)

where the trade-off parameter *t* > 0 controls the proportion of spatial information, and *x*_*ij*_ and *x*_*pq*_ are normalized to ${\tilde{x}}_{ij}$ and ${\tilde{x}}_{pq}$, respectively. These factors affect the value of the reconstruction weight *W*.

**Fig 2:**
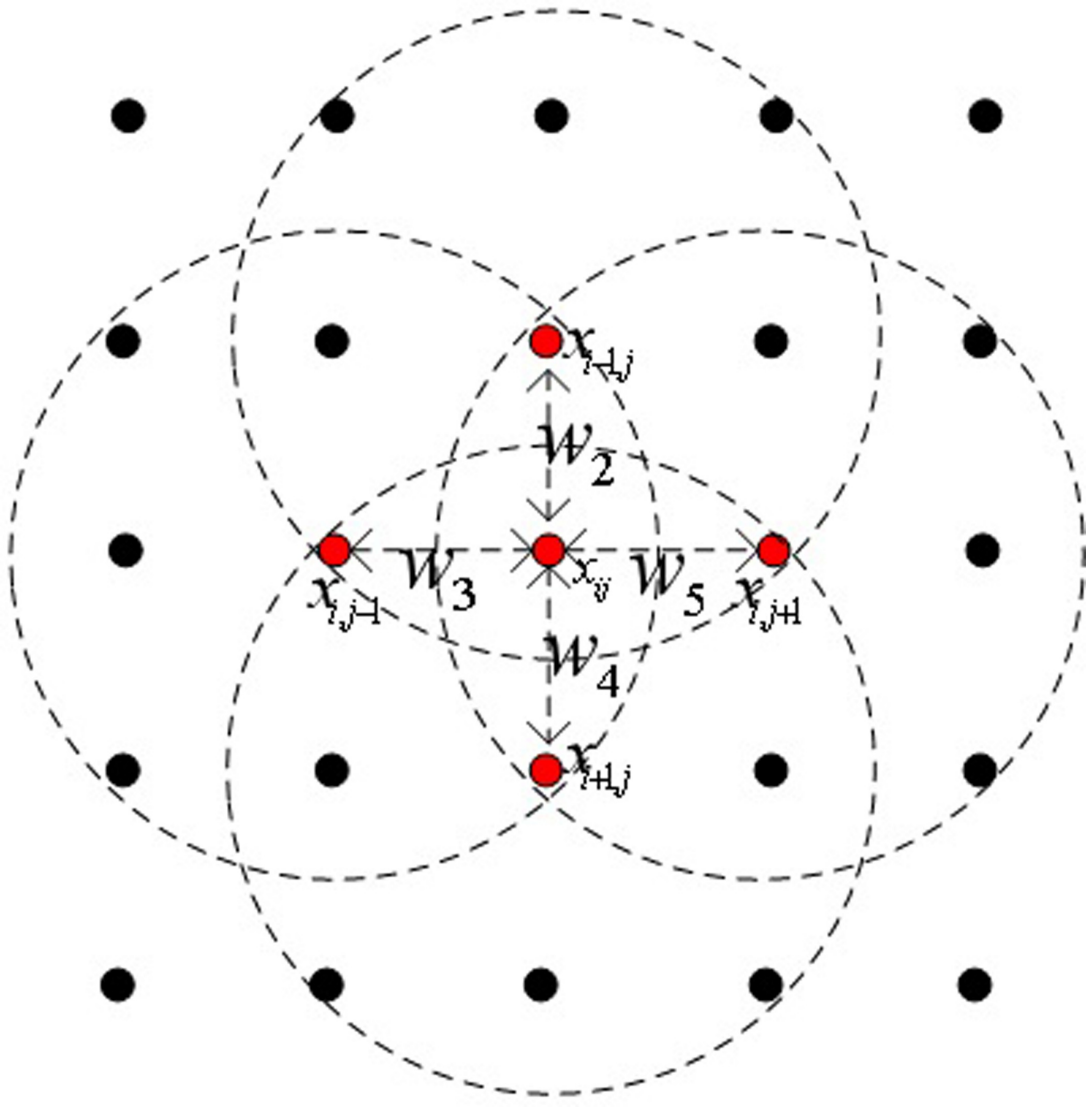
Four nearest neighbors of a pixel *x*_*ij*_ and their weights between *x*_*ij*_.

Using the weights obtained, we constructed the weighted Laplacian vector. Let *x*_*st*_ be the four nearest neighbors of *x*_*ij*_, where *s* = *i*– 1, *i* + 1; *t* = *j*– 1, *j* + 1, as shown in Fig 2.

Let be *α*_*ij*_ be the sparse vector associated with *x*_*ij*_ (i.e., *Dα*_*ij*_ = *x*_*ij*_). Then, we construct the weighted Laplacian vector at the pixel *x*_*ij*_ as:

$$\begin{array}{l} \nabla^{2}(x_{ij})=w_{1}x_{ij}-w_{2}x_{i-1,j}-w_{3}x_{i,j-1}-w_{4}x_{i+1,j}-w_{5}x_{i,j+1}\\ \;=D(w_{1}\alpha_{ij}-w_{2}\alpha_{i-1,j}-w_{3}\alpha_{i,j-1}-w_{4}\alpha_{i+1,j}-w_{5}\alpha_{i,j+1}) \end{array}$$ (9)

where $w_{1}=\Sigma_{i=2}^{5}w_{i}$.

To incorporate the smoothness across the neighboring spectral pixels, *∇*^2^(*x*_*ij*_) is set to zero, based on which a new large block sparse dictionary for SOMP is constructed with six times as many rows and five times as many columns as the original dictionary. Taking the Indian pines dataset with 10% training samples as an example, the dimension of the original dictionary was 200 × 1027, and the block sparse matrix dimension we built was 1200 × 5135. Then, the optimization problem in (1) can be redefined as a new sparse recovery problem with the Laplacian smoothing constraint, and it is formulated as:

$$\min\;{\left\|\tilde{\alpha }\right\|}_{0},\;\text{S}.\text{t}.\;D_{L\;arg\;e\;BlockSparse}\tilde{\alpha }=\tilde{x}$$ (10)

where

$$D_{L\;arg\;e\;BlockSparse}\left[\begin{array}{lllll} w_{1}D & -w_{2}D & -w_{3}D & -w_{4}D & -w_{5}D\\ D & 0 & 0 & 0 & 0\\ 0 & D & 0 & 0 & 0\\ 0 & 0 & D & 0 & 0\\ 0 & 0 & 0 & D & 0\\ 0 & 0 & 0 & 0 & D \end{array}\right]$$ (11)

$$\tilde{\alpha }=\left[\begin{array}{l} \alpha_{ij}\\ \alpha_{i-1,j}\\ \alpha_{i,j-1}\\ \alpha_{i+1,j}\\ \alpha_{i,j+1} \end{array}\right]$$ (12)

$$\tilde{x}=\left[\begin{array}{l} \begin{array}{l} 0\\ x_{ij} \end{array}\\ x_{i-1,j}\\ x_{i,j-1}\\ x_{i+1,j}\\ x_{i,j+1} \end{array}\right]$$ (13)

$$w_{1}=\Sigma_{i=2}^{5}w_{i}$$ (14)

Normally, we can assign, *w*_1_ = 1, and *w*_*i*_ (i = 2, …,5) are normalized as ${\tilde{w}}_{i}=\frac{w_{i}}{\partial }$, where $\partial =\sum_{i=2}^{5}w_{i}$.

For the pixels at the center, all weights are present. However, for the pixels on the edge or corner, some weights will not be present, which will cause an imbalance. To avoid the imbalance, we assign *w*_*i*_ = 0.25 (i = 2, …,5).

We all pointed out that the L0 norm is a non-deterministic polynomial hard (NP-hard) problem, while the L1 norm is the optimal convex approximation of the L0 norm, and the L1 norm is easier to solve than the L0 norm. Additionally, the equality constraints in (11) cannot be satisfied completely, it allows approximation error, thus the problem can be written as:

$$min\;{\left\|\tilde{\alpha }\right\|}_{1}+\frac{\gamma }{2}{\left\|\tilde{x}-\;D_{L\;arg\;e\;BlockSparse}\tilde{\alpha }\right\|}_{2}^{2},$$ (15)

where γ denotes the regularization parameter.

The problem in (15) is a standard sparse recovery problem, and SOMP can be implemented to solve it. Once the problem in (15) is solved, the total class-dependent reconstruction residuals between the original test samples and the approximations obtained from each of the *K* class sub-dictionaries can be calculated as:

$$r_{k}(x)=\sqrt{\sum_{i=1}^{5}{\left\|x_{st}-D_{L\;arg\;e\;BlockSparse_{i}}^{k}{\tilde{\alpha }}_{st}^{k}\right\|}_{2}^{2}}$$ (16)

where *k* ∈ *κ* = {1,2,3,…,*K*}, *s* = *i*– 1, *i* +1; *t* = *j*– 1, *j* + 1. *x* represents a concatenation of the five pixels, *x*_*ij*_, *x*_*i*– 1,*j*_, *x*_*i*,*j*−1_, *x*_*i*+1,*j*_*x*_*i*,*j*+1_, as shown in Fig 2, and ${\tilde{\alpha }}_{st}^{k}$ denotes the portion of the recovered sparse vector *x*_*st*_ associated with the kth-class subdictionary, $D_{L\;arg\;e\;BlockSparse_{i}}^{k}$. The test sample is *x*_*ij*_ assigned to the class that minimizes the residual:

$$identity(x_{ij})=arg\;\underset{k=1,2,\ldots ,K}{min}r_{k}(x)$$ (17)

## Experiments and discussion

### Datasets

Three well-known publicly available HSI datasets, namely the Indian Pines, University of Pavia, and Salinas, were used to evaluate the performance of WLSC-SR in this study. The number of samples in the Indian Pines, Pavia University, and Salinas scene images are shown in Table 1, in which the background color was used to distinguish different classes.

**Table 1:** Number of samples in the Indian Pines, Pavia University, and Salinas scene image.

Indian Pines			Pavia University			Salinas scene		
#	Class Names	SampleNumber	#	Class Names	SampleNumber	#	Class Names	SampleNumber
1	Alfalfa	46	1	Asphalt	6631	1	Brocoli_green_weeds_1	2009
2	Corn-notill	1428	2	Meadows	18649	2	Brocoli_green_weeds_2	3726
3	Corn-mintill	830	3	Gravel	2099	3	Fallow	1976
4	Corn	237	4	Trees	3064	4	Fallow_rough_plow	1394
5	Grass-pasture	483	5	Painted metal sheets	1345	5	Fallow_smooth	2678
6	Grass-trees	730	6	Bare Soil	5029	6	Stubble	3959
7	Grass-pasture-mowed	28	7	Bitumen	1330	7	Celery	3579
8	Hay-windrowed	478	8	Self-Blocking Bricks	3682	8	Grapes_untrained	11271
9	Oats	20	9	Shadows	947	9	Soil_vinyard_develop	6203
10	Soybean-notill	972				10	Corn_senesced_green_weeds	3278
11	Soybean-mintill	2455				11	Lettuce_romaine_4wk	1068
12	Soybean-clean	593				12	Lettuce_romaine_5wk	1927
13	Wheat	205				13	Lettuce_romaine_6wk	916
14	Woods	1265				14	Lettuce_romaine_7wk	1070
15	Buildings-Grass-Trees-Drives	386				15	Vinyard_untrained	7268
16	Stone-Steel-Towers	93				16	Vinyard_vertical_trellis	1807
Total Number		10249	Total Number		42776	Total Number		54129

Indian Pines: This scene was gathered by the AVIRIS sensor over the Indian Pines test site in northwestern Indiana and consists of 145 × 145 pixels and 224 spectral reflectance bands in the wavelength range 0.4 to 2.5 μm. This scene, which includes 16 different ground-truths, contains two-thirds of agriculture and one-third of forest or other natural perennial vegetation. The number of bands was reduced to 200 by removing the 24 water absorption bands.

Pavia University: This scene was acquired by the ROSIS sensor during a flight campaign over Pavia, northern Italy. The number of spectral bands was 103 at Pavia University. It is a 610 × 340 pixel image containing nine different ground objects with a geometric resolution of 1.3 meters.

Salinas: This scene was collected by the 224-band AVIRIS sensor over the agricultural area of Salinas Valley, California and is characterized by high spatial resolution (3.7-m pixels). After discarding 20 water absorption bands, the size of this data image was 512 × 217, with 204 bands. Salinas ground-truth contains 16 classes, including vegetables, bare soils, and vineyard fields.

### Quantitative metrics

Normally, overall accuracy (OA), average accuracy (AA), class accuracy (CA), and Kappa coefficient are adopted to evaluate the quality of the classification results of HSI.OA refers to the ratio between the number of correctly classified categories and the total number of categories, AA represents the mean of the percentage of correctly classified pixels for each class, CA measures the separate classification accuracy of various ground objects in the dataset, and the Kappa coefficient estimates the percentage of classified pixels corrected by the number of agreements that would be expected purely by chance. It is believed that the classification performance of the classifier is good when the Kappa coefficient is greater than 0.75. However, when the Kappa coefficient is less than 0.40, the performance is poor [10, 42].

### Parameter analysis

**Fig 3:**
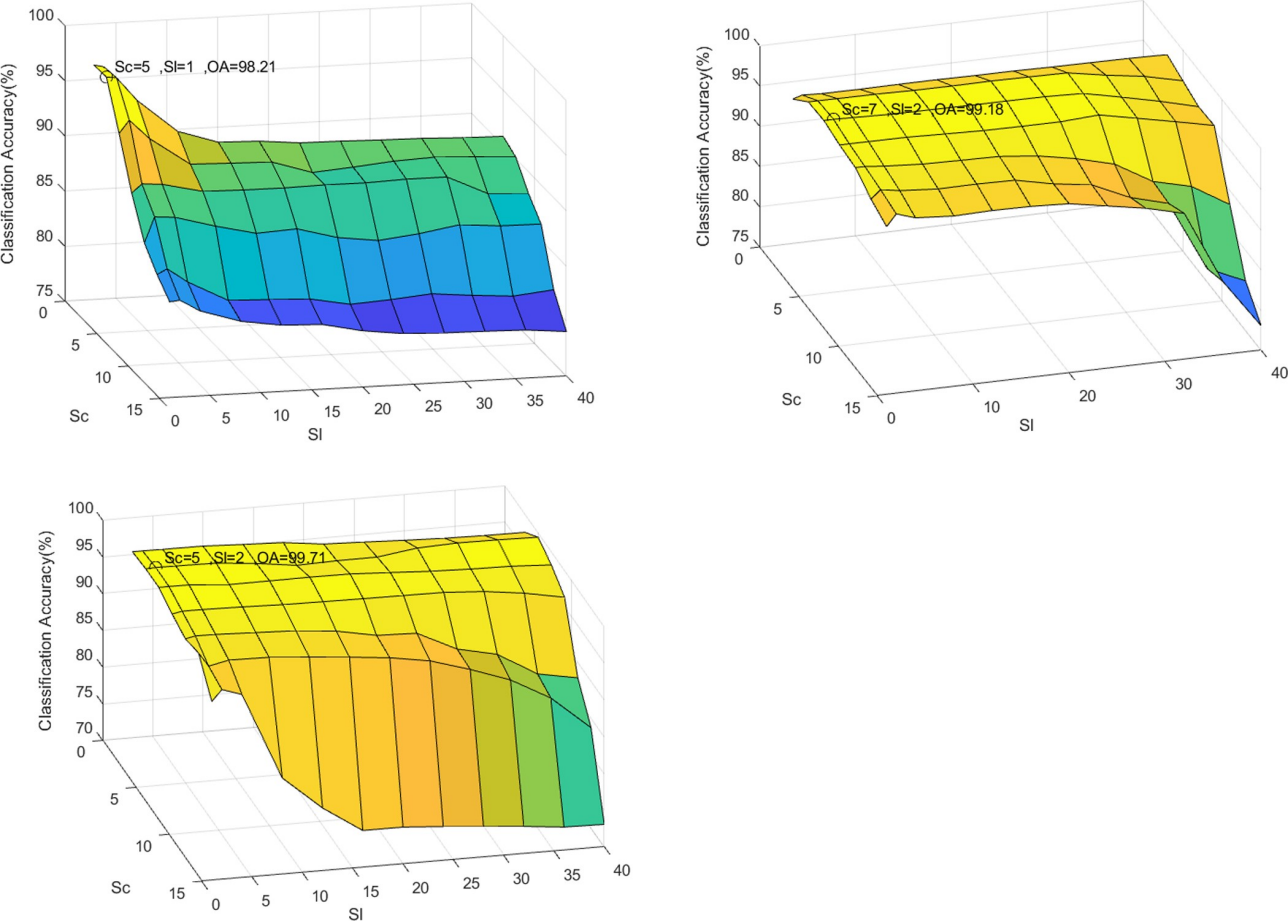
Effect of Sl and Sc. (a) Indian Pines, (b) Pavia University, and (c) Salinas Scene.

In the proposed classification method, there are two primary impact parameters: the sparsity level Sl and the WLSC-SR model test region scale Sc, which can affect the classification performance from different aspects. Experiments on the Indian Pines, Pavia University, and Salinas showed the OAs of different Sl and Sc, based on which the optimal parameters were determined. Fig 3A–3C show the effects of Sl and Sc in the three datasets, respectively. The optimal classification result is shown in the graph.

As shown in Fig 3, when the value of the test region scale Sc is fixed, the OA for Indian Pines, Pavia University, and Salinas Scene can consistently achieve the best performance when the sparsity level Sl is 1 or 2. As Sl increases, the solution of (16) converges to the pseudo inverse solution, which is no longer sparse, which deteriorates the classification performance. Additionally, when the sparsity level Sl is small, if Sc is too large, the neighboring pixels cannot be faithfully approximated by a few training samples. In other words, the OA is reduced. Moreover, the value of Sc for a large dataset is relatively large, and vice versa. For example, when Sl for Indian Pines, Pavia University, and Salinas Scene are 1, 2, and 3, respectively, the OA can obtain the best value when Sc is 5, 7, and 5, respectively. On the contrary, the experimental results show that when Sc is equal to 40, the classification performance is far worse than the best value.

Additionally, since the Pavia University image is larger than the Indian Pines and Salinas Scene image, OA obtains the best value when Sc = 7 in Pavia University image classification. For Indian Pines and Salinas Scene images, the corresponding Sc is equal to 5.

### Comparison of different classifiers

In this section, the proposed methods are compared with the SVM method [5], JSR classification method [17], SR classification method [16], and sparse representation nearest neighbor (NN-SR) classification method [18]. Additionally, the original nearest neighbor classification methods, such as multiscale adaptive sparse representation (MASR) [19] as well as the joint sparse representation joint nearest neighbor(JNN-JSR) [27], are also compared with the joint sparse representation weighting joint nearest neighbor method (WJNN-JSR) [27]. These classification methods were implemented using optimal parameters.

**Fig 4:**
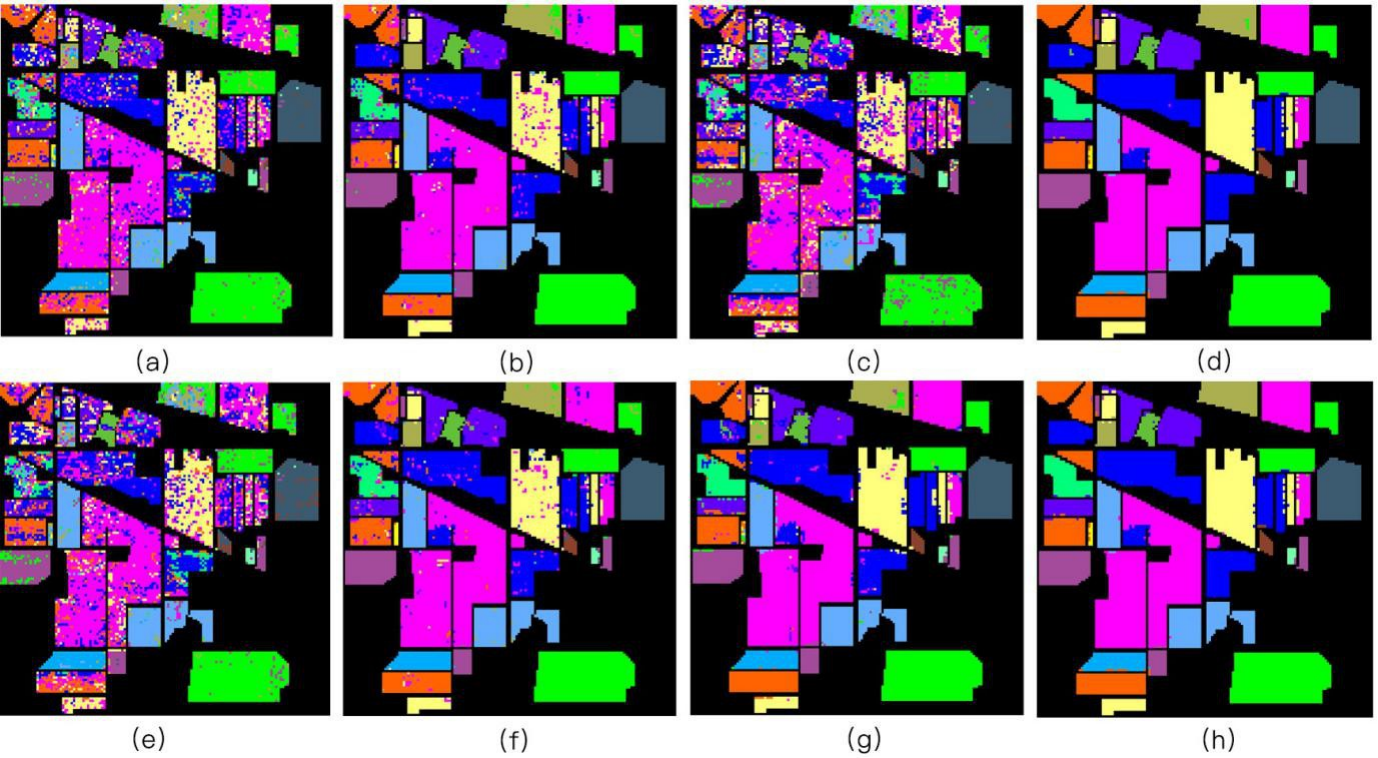
The Indian Pines image. (a) SVM [1] (OA = 72.26%); (b) JSR [17] (OA = 91.71%); (c) SR [16] (OA = 63.89%); (d) MASR [19] (OA = 96.91%); (e) NN-SR [18] (OA = 65.44%); (f) JNN-JSR [27] (OA = 93.12%); (g) WJNN-JSR [27] (OA = 93.65%); (h) WLSC-SR (OA = 98.21%).

**Fig 5:**
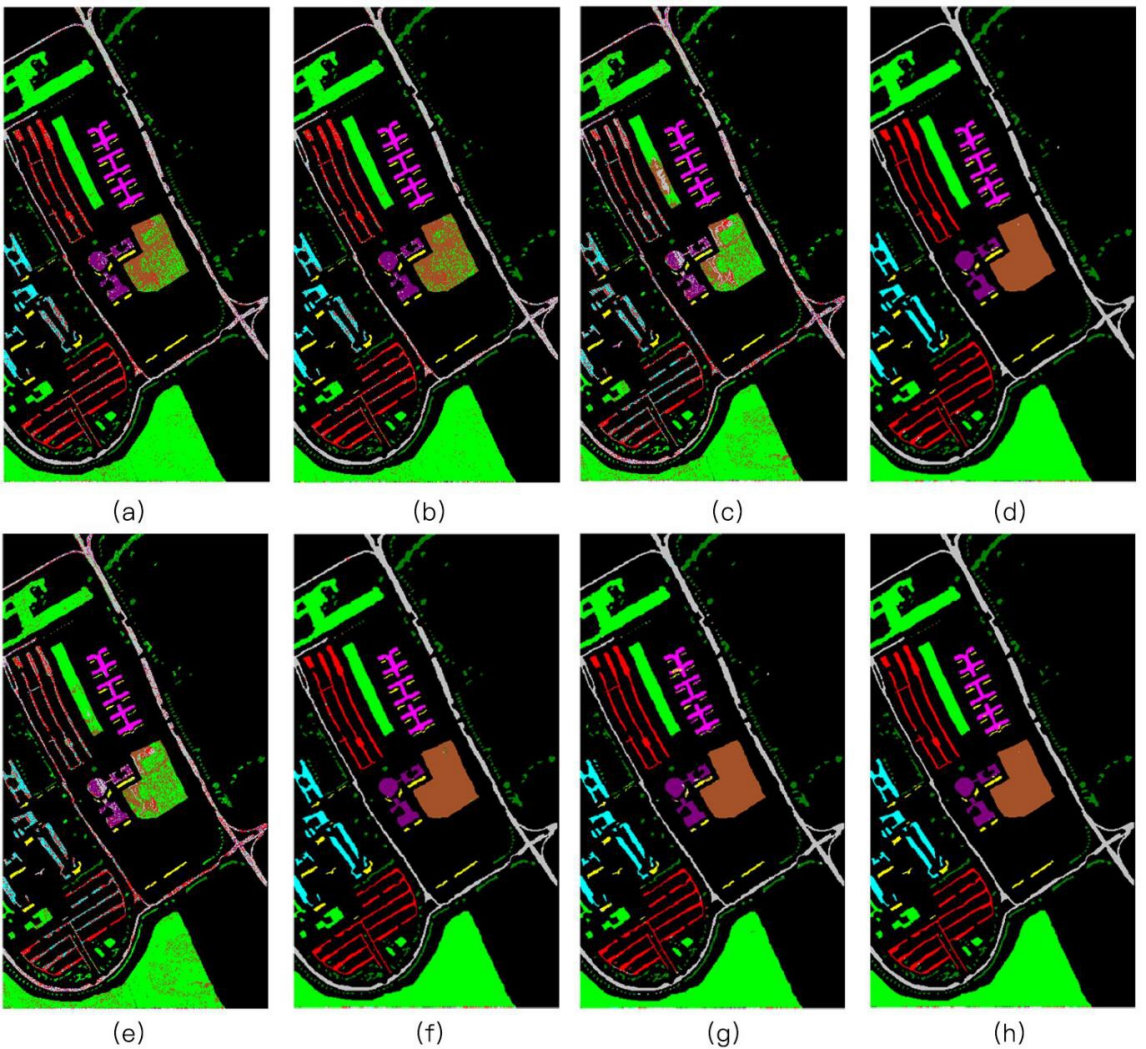
The Pavia University image. (a) SVM [1] (OA = 85.42%); (b) JSR [17] (OA = 89.31%); (c) SR [16] (OA = 72.01); (d) MASR [19] (OA = 88.01%); (e) NN-SR [18] (OA = 73.27%); (f) JNN-JSR [27] (OA = 96.60%); (g) WJNN-JSR [27] (OA = 97.42%); (h) WLSC-SR (OA = 98.18%).

**Fig 6:**
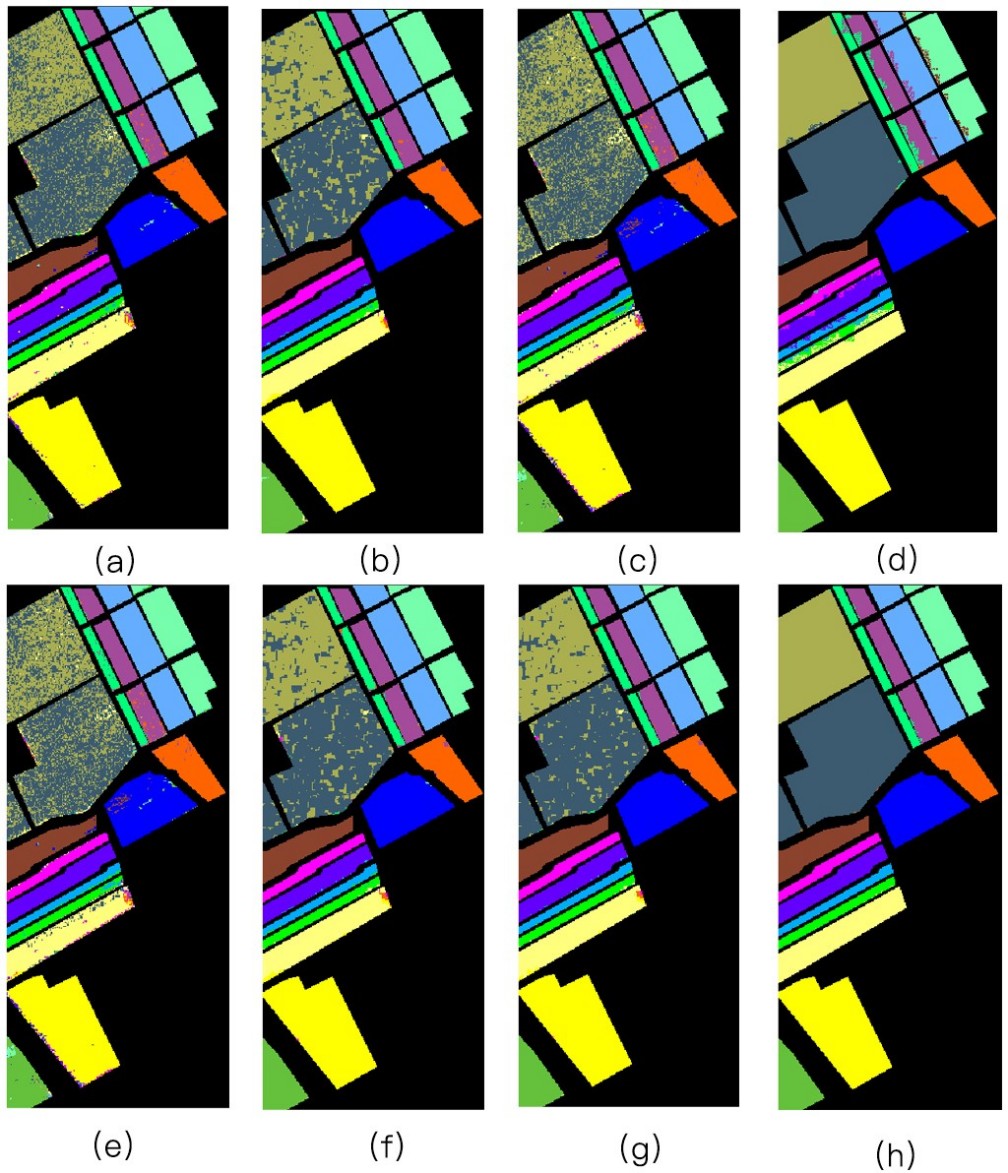
The Salinas image. (a) SVM [1] (OA = 88.67%); (b) JSR [17] (OA = 92.99%); (c) SR [16] (OA = 85.09); (d) MASR [19] (OA = 93.43%); (e) NN-SR [18] (OA = 85.37%); (f) JNN-JSR [27] (OA = 94.46%); (g) WJNN-JSR [27] (OA = 95.61%); (h) WLSC-SR (OA = 99.71%).

Three different experiments were conducted on three different datasets: Indian Pines, Pavia University, and Salinas. For each class of every dataset, 30% of the labeled pixels were randomly sampled for training, while the remaining 70% were used to test the classifiers. Figs 4–6 illustrate different classification maps obtained by different methods on different datasets.

The first experiment was performed using the Indian Pines dataset. Table 2 shows the classification performance with the corresponding OA, AA, and Kappa values. The bold values indicate the best classification accuracy. As can be observed, the classification maps of the SVM and SR methods have a very noisy appearance. By considering the spatial context, the JSR, MASR, NN-SR, and WJNN-JSR algorithms can deliver a comparatively smooth result but fail to detect meaningful regions. Although the JNN-JSR algorithm shows improvements in detecting the details, some noisy behavior will exist on the obtained classification maps for these approaches.

**Table 2:** Classification accuracy (in percent) of the Indian Pines in the SVM [1], JSR [17], SR [16], MASR [19], NN-SR [18], JNN-JSR [27], WJNN-JSR [27], and WLSC-SR.

#	SVM	JSR	SR	MASR	NN-SR	JNN-JSR	WJNN-JSR	WLSC-SR
1	81.25	85.42	43.90	78.05	74.07	**98.15**	90.57	96.77
2	86.28	94.88	63.34	96.19	66.67	92.96	96.96	**98.20**
3	72.80	94.93	51.27	95.18	63.55	94.96	**99.95**	98.62
4	58.10	91.43	37.27	90.14	51.28	85.90	96.73	**96.97**
5	92.39	89.49	82.94	98.16	90.34	95.99	**100.00**	98.23
6	96.88	98.51	91.62	99.54	97.46	**99.60**	96.18	99.22
7	43.48	91.30	67.60	**100.00**	76.92	99.18	88.14	84.21
8	98.86	99.55	94.62	99.30	97.96	**100.00**	96.65	**100.00**
9	50.00	0.00	34.44	50.00	30.00	45.00	94.10	**94.29**
10	71.53	89.44	63.76	95.66	78.51	93.49	**99.94**	97.94
11	84.38	97.34	71.12	97.87	78.08	96.72	95.03	**98.14**
12	85.51	88.22	42.47	92.88	65.31	93.81	95.81	**97.84**
13	**100.00**	**100.00**	91.19	90.76	98.58	96.79	92.61	92.25
14	93.30	99.14	89.75	**99.56**	90.80	99.30	91.86	99.43
15	64.91	99.12	36.13	**99.42**	78.95	94.47	93.13	99.26
16	88.24	96.47	88.21	**100.00**	90.53	96.84	94.54	92.42
OA	72.26	91.71	63.89	96.91	65.44	93.12	93.65	**98.21**
AA	73.36	94.20	68.94	92.67	69.25	94.32	**95.48**	94.61
Kappa	73.86	90.39	58.66	96.47	60.34	93.02	92.76	**97.96**

In contrast, the proposed WLSC-SR algorithm has a limited improvement in the average classification accuracy, denoising, and misclassification at the edges of the data, and the overall scene is significantly reduced. Therefore, according to the classification results, the proposed method still has advantages in terms of OA and kappa values. For example, compared with other methods, the WLSC-SR algorithm achieves the highest classification accuracy in classes 2, 4, 8, 9, 11, and 12. Additionally, OA and Kappa reached their highest values. The second and third experiments were conducted on the Pavia University and Salinas datasets, respectively. The training sample selection was the same as in the first experiment.

Table 3 presents the classification performance with the corresponding OA, AA, and Kappa values for Pavia University. As shown in the table, the proposed WLSC-SR algorithm obtains higher accuracy than the other compared methods in terms of OA, AA, and Kappa. These spectral-spatial joint algorithms, such as JSR, MASR, NN-SR, JNN-JSR, WJNN-JSR, and WLSC-SR, perform better than SVM and SR which only use spectral information. For example, the OA of the SR algorithm is only 72.01%, and compared with the SR algorithm, the OA of the WJNN-JSR and WLSC-SR algorithms were improved by 25.41% and 27.17%, respectively.

**Table 3:** Classification accuracy (in percent) of the Pavia University in the SVM [1], JSR [17], SR [16], MASR [19], NN-SR [18], JNN-JSR [27], WJNN-JSR [27], and WLSC-SR.

#	SVM	JSR	SR	MASR	NN-SR	JNN-JSR	WJNN-JSR	WLSC-SR
1	95.61	71.60	57.98	82.46	57.75	98.88	98.84	**99.96**
2	94.95	91.06	73.02	96.15	75.55	96.62	95.84	**98.90**
3	69.93	95.63	64.16	72.91	70.65	97.04	97.32	**100.00**
4	76.19	95.55	90.01	90.82	89.49	86.44	98.00	**98.65**
5	94.89	99.05	99.5	98.51	99.33	**99.90**	99.88	97.77
6	69.22	96.60	63.11	70.82	60.97	98.24	97.64	**99.97**
7	60.03	97.98	86.33	84.10	88.05	99.16	**99.51**	99.14
8	82.64	92.07	68.86	82.04	74.53	99.17	98.71	**99.26**
9	**99.92**	69.4	96.01	96.07	96.44	98.86	99.17	96.37
OA	85.42	89.31	72.01	88.01	73.27	96.60	97.42	**99.18**
AA	82.60	89.88	78.15	85.99	79.60	97.15	98.32	**98.89**
Kappa	81.08	86.07	64.23	83.97	65.69	95.88	96.27	**98.91**

Table 4 presents the classification performance with the corresponding OA, AA, and Kappa values for the Salinas image. It can be seen that because WJNN-JSR effectively utilizes multi-scale spatial information through an adaptive sparse strategy, the AA of WJNN-JSR has been significantly improved, but some noise still exists around the boundary of different ground objects, so the improvement of OA and Kappa is limited. By contrast, considering the boundaries of adjacent ground objects in the image, the OA, AA, and Kappa of our proposed WLSC-SR method were improved by 4.1%, 2.89%, and 4.8%, respectively.

**Table 4:** Classification accuracy (in percent) of the Salinas scene in the SVM [1], JSR [17], SR [16], MASR [19], NN-SR [18], JNN-JSR [27], WJNN-JSR [27], and WLSC-SR.

#	SVM	JSR	SR	MASR	NN-SR	JNN-JSR	WJNN-JSR	WLSC-SR
1	99.65	**100.00**	99.06	99.72	98.97	**100.00**	**100.00**	**100.00**
2	99.44	99.97	98.62	99.81	98.42	99.97	**100.00**	**100.00**
3	94.33	99.39	97.25	99.42	97.64	**100.00**	**100.00**	**100.00**
4	97.55	95.37	99.46	98.57	**99.54**	90.85	95.95	99.28
5	98.39	98.38	94.16	**98.45**	94.61	98.28	91.52	97.65
6	99.98	99.92	99.50	99.82	99.51	99.90	**100.00**	99.89
7	98.94	99.75	99.38	98.92	99.22	**99.91**	99.43	99.60
8	78.94	86.07	61.06	86.64	62.75	73.83	84.48	**99.87**
9	99.33	99.74	96.84	99.17	96.66	99.90	**100.00**	**100.00**
10	85.40	93.63	89.31	95.47	90.17	99.07	99.69	**99.91**
11	90.84	**99.72**	98.51	99.20	98.73	99.60	98.81	99.20
12	96.99	98.48	99.92	**100.00**	99.83	98.66	92.77	99.93
13	97.02	92.96	98.12	92.50	97.57	97.66	**99.18**	98.58
14	91.01	98.21	93.34	94.39	94.38	**99.90**	86.93	98.26
15	64.73	65.56	64.44	78.30	64.49	80.09	91.40	**99.94**
16	97.52	98.38	97.73	99.76	97.81	**100.00**	**100.00**	**100.00**
OA	88.67	91.54	85.09	93.43	85.37	94.46	95.61	**99.71**
AA	93.13	95.35	92.92	96.26	93.12	96.56	96.62	**99.51**
Kappa	87.39	90.57	83.43	92.68	83.75	93.61	94.88	**99.68**

### Computational complexity

Experiments were performed using MATLAB 2018b on a computer with an Intel-2.60GHz CPU, 16GB memory, and a 64-bit Windows 7 system. On three real HSI datasets, complete execution of our algorithm may take several minutes to several hours, but the other compared methods in this study do not take that long. Specifically, the main computational cost of this method is the operation of weighted parameters and the large block sparse dictionary in SOMP. With the development of computing hardware and cloud computing technology, we believe that the consumption time will be significantly reduced. Additionally, the ideal parameters or hyperparameters used by the various algorithms for the results are listed in Table 5.

**Table 5:** The ideal parameters or hyperparameters used by the various algorithms.

methods	parameters or hyperparameters
SVM	the width of Gaussian Kernel *γ* ∈ [0,1,3], regularization parameter C = 40
JSR	Sparsity level k ∈ [5,10],weight factor γ∈[0.01,10], neighborhood size W = 5
SR	Sparsity level k ∈ [3,5,7,9]
MASR	Sparsity level K = 3, neighborhood size W ∈ {3×3,5×5,7×7,9×9,11×11,13×13,15×15}
NN-SR	Sparsity level S = 10, neighborhood size W ∈{41×41}
JNN-JSR	neighborhood size W = 5, iteration upper boundary M = 100, the nearest rang NR = 1
WJNN-JSR	neighborhood size W = 3, Sparsity level K = 5, the stopping criterion σ = 0.01
WLSC-SR	neighborhood size *S*_*c*_ = {5×5,7×7},sparsity level *S*_*l*_ = {1,2}

## Conclusions

In this context, we proposed a new classification method for HSI. The proposed WLSC-SR strengthens the spatial information between the center pixel and its four nearest neighborpixels by constructing a smoothing constraint Laplacian vector. The vector can overcome the boundary characteristics of adjacent ground objects in the HSI. Experiments on three real HSI datasets revealed that the proposed WLSC-SR method outperforms several other well-known classifiers in terms of OA, AA, Kappa, and visual comparison of classification maps. Finally, we verified the effectiveness and superiority of WLSC-SR. Another method that the authors will explore in future work to further improve the classification accuracy is employing discriminative learning algorithms and optimizing the dictionary structure. Therefore, the focus of our future research is to explore more efficient solutions to optimize this method.
